# Identification of a fibrinogen-related protein (FBN9) gene in neotropical anopheline mosquitoes

**DOI:** 10.1186/1475-2875-10-21

**Published:** 2011-02-02

**Authors:** Sabrina B Oliveira, Izabela C Ibraim, Wanderli P Tadei, Jeronimo C Ruiz, Laila A Nahum, Cristiana FA Brito, Luciano A Moreira

**Affiliations:** 1 Laboratório de Malária, Centro de Pesquisas René Rachou, Fundação Oswaldo Cruz - FIOCRUZ, Belo Horizonte, MG 30190-002, Brazil; 2 Laboratório de Malária e Dengue, Instituto Nacional de Pesquisas da Amazônia - INPA, Manaus, AM 69060-000, Brazil; 3 Laboratório de Parasitologia Celular e Molecular, Centro de Pesquisas René Rachou, Fundação Oswaldo Cruz - FIOCRUZ, Belo Horizonte, MG 30190-002, Brazil; 4 Centro de Excelência em Bioinformática, Fundação Oswaldo Cruz - FIOCRUZ, Belo Horizonte, MG 30190-110, Brazil

## Abstract

**Background:**

Malaria has a devastating impact on worldwide public health in many tropical areas. Studies on vector immunity are important for the overall understanding of the parasite-vector interaction and for the design of novel strategies to control malaria. A member of the fibrinogen-related protein family, *fbn9*, has been well studied in *Anopheles gambiae *and has been shown to be an important component of the mosquito immune system. However, little is known about this gene in neotropical anopheline species.

**Methods:**

This article describes the identification and characterization of the *fbn9 *gene partial sequences from four species of neotropical anopheline primary and secondary vectors: *Anopheles darlingi, Anopheles nuneztovari, Anopheles aquasalis*, and *Anopheles albitarsis *(namely *Anopheles marajoara*). Degenerate primers were designed based on comparative analysis of publicly available *Aedes aegypti *and *An. gambiae *gene sequences and used to clone putative homologs in the neotropical species. Sequence comparisons and Bayesian phylogenetic analyses were then performed to better understand the molecular diversity of this gene in evolutionary distant anopheline species, belonging to different subgenera.

**Results:**

Comparisons of the *fbn9 *gene sequences of the neotropical anophelines and their homologs in the *An. gambiae *complex (Gambiae complex) showed high conservation at the nucleotide and amino acid levels, although some sites show significant differentiation (non-synonymous substitutions). Furthermore, phylogenetic analysis of *fbn9 *nucleotide sequences showed that neotropical anophelines and African mosquitoes form two well-supported clades, mirroring their separation into two different subgenera.

**Conclusions:**

The present work adds new insights into the conserved role of *fbn9 *in insect immunity in a broader range of anopheline species and reinforces the possibility of manipulating mosquito immunity to design novel pathogen control strategies.

## Background

Mosquito-borne diseases, including malaria and arboviruses, such as dengue, depend on complex interactions among pathogens, insect vectors, and hosts. Studies of vector immunity are of particular importance to understanding these complex interactions and could lead to the development of novel disease control strategies [1-6]. Vectorial competence, which refers to the ability of arthropods to acquire, maintain, and transmit microbial agents [7], is directly related to insect immunity. Several immunity-related genes have been identified in Old World vectors [8-10]. However, related studies in neotropical anopheline species are still incipient.

Among the important immunity genes are the ones enconding members of the fibrinogen-related protein family (FREP or FBN), which are pattern recognition receptors and have been considered as promising candidates for parasite control strategies [10-12]. Within this family, the *fbn*9 gene was found to be upregulated when *Anopheles gambiae *mosquitoes were fed on blood infected with parasites (*Plasmodium falciparum*) or bacteria (*Escherichia coli *or *Staphylococcus aureus) *[10]. Furthermore, when this gene was knocked-down, parasite loads significantly increased [10]. More recently, the *fbn9 *gene was found to be conserved among members of the *An. gambiae *complex [13]. The *An. gambiae *complex comprehends seven closely related species (*An. gambiae*, *Anopheles arabiensis*, *Anopheles melas, Anopheles merus*, *Anopheles bwambae*, and *Anopheles quadriannulatus *A and B), from which *An. gambiae *and *An. arabiensis *have been described as important vectors of human malaria [14].

In South America, the most important malaria vectors comprise the *Anopheles *(*Nyssorhynchus*) *darlingi, Anopheles albimanus*, and members of the *Anopheles albitarsis *complex (*An. albitarsis, Anopheles oryzalimnetes, Anopheles marajoara, Anopheles deaneorum, Anopheles janconnae*, and *An. albitarsis *F) [15]. Secondary vectors include *Anopheles nuneztovari*, *Anopheles aquasalis *and other members of the same subgenera [16-19]. In Brazil, of the 54 anopheline species that have been recorded, 13 were found to be naturally infected with *Plasmodium *spp. [16,20], emphasizing the importance of further vector/parasite studies, including laboratory-based infections.

The identification and characterization of the *fbn9 *gene partial sequences from four species of neotropical anopheline mosquitoes has been performed in this study, followed by comparisons to sequences of the *An. gambiae *complex available in public databases. Further comparisons of synonymous (silent) and non-synonymous (changing) substitution rates in its amino acid sequence have been applied to try understanding its molecular evolution. This study allowed a better understanding of the molecular diversity and predicted function of this immunity gene in a broader range of mosquito species.

## Methods

### Mosquito collection and identification

Mosquitoes were collected from different locations (60-70 specimens from each locality; Table 1). *Anopheles darlingi *specimens were collected from larval breeding sites and by capturing adults through traps in rural areas of Manaus (Amazon, Brazil). Larvae were also collected and maintained in rearing conditions and adults identified upon emergence. *Anopheles albitarsis *samples were also collected in Porto Velho (Rondonia, Brazil). *Anopheles aquasalis *was obtained from a laboratory colony reared at 27°C, 80% humidity and 12h L:D cycle maintained at FIOCRUZ, Belo Horizonte-MG, Brazil. Mosquitoes were individually identified according to their morphology through taxonomic keys, which are based on particularities on their tarsi, abdomen, and wing veins [21]. The identification of specimens of the *An. albitarsis *complex was confirmed by sequencing the internal transcribed spacer 2, ITS2, as described below.

**Table 1:** Sequences used for the FBN9 phylogenetic analysis.

Accession Number	Gi	Sequence	Species	Origin
HQ188296	312145129	Contig2-fbn-aqua	*An. aquasalis*	Brazil (insectary, CPqRR)
HQ188298	312145133	Contig3-fbn-aqua	*An. aquasalis*	Brazil (insectary, CPqRR)
HQ188295	312145127	Contig21-fbn-darl	*An. darlingi*	Brazil (Manaus, AM^a^)
HQ188300	312145137	Contig7-fbn-darl	*An. darlingi*	Brazil (Manaus,AM)
HQ188297	312145131	Contig2-fbn-albit	*An. marajoara*	Brazil (Manaus, AM)
HM623677	312145141	Contig14-fbn-albit	*An. marajoara*	Brazil (Manaus, AM)
HM635775	312145143	Contig1-fbn-albit	*An. marajoara*	Brazil (Manaus, AM)
HQ188299	312145135	Contig5-fbn-albit.ro	*An. marajoara*	Brazil (Porto Velho, RO)
HQ188294	312145125	Contig19-fbn-nunezv2	*An. nuneztovari*	Brazil (Manaus, AM)
HQ188301	312145139	Contig8-fbn- nunezv	*An. nuneztovari*	Brazil (Manaus, AM)

EU304626.1	167861637	ARA087_B	*An. arabiensis*	Cameroon (Kousseri)
EU304631.1	167861647	ARA125_A	*An. arabiensis*	Cameroon (Kousseri)
EU304652.1	167861689	GAM72_A	*An. gambiae*	Cameroon (Mbebé and Nyabéssan)
EU304650.1	167861685	GAM69_A	*An. gambiae*	Cameroon (Mbebé and Nyabéssan)
EU304649.1	167861683	GAM66	*An. gambiae*	Cameroon (Mbebé and Nyabéssan)
EU304647.1	167861679	GAM15_A	*An. gambiae*	Cameroon (Mbebé and Nyabéssan)
EU304646.1	167861677	GAM13_B	*An. gambiae*	Cameroon (Mbebé and Nyabéssan)
EU304644.1	167861673	GAM07_B	*An. gambiae*	Cameroon (Mbebé and Nyabéssan)
EU304682.1	167861749	QUA24_B	*An. quadriannulatus*	South Africa (Kruger National Park)
EU304675.1	167861735	QUA16	*An. quadriannulatus*	South Africa (Kruger National Park)
EU304670.1	167861721	MER563_A	*An. merus*	Mozambique (Furvela)
EU304668.1	167861721	MER562_A	*An. merus*	Mozambique (Furvela)
EU304658.1	167861701	MEL22	*An. melas*	Cameroon (Ipono)
EU304656.1	167861697	MEL15	*An. melas*	Cameroon (Ipono)
EU304639.1	167861663	BWA17	*An. bwambae*	Uganda (Bwamba)
EU304641.1	167861667	BWA18_B	*An. bwambae*	Uganda (Bwamba)

^a^AM - Amazon State, RO - Rondonia State, Brazil.

### ITS2 genotyping

Genomic DNA has been isolated from pools of 10 mosquitoes following previously published protocol [22]. PCR reactions were performed using the primers CP16 (5'-GCGGGTACCATGCTTAAATTTAGGGGGTA-3') and CP17 (5'-GCGCCGCGGTGTGAACTGCAGGACACATG-3') [23]. Each reaction contained 0.2 μM of each primer, 0.2 mM dNTPs, buffer (50 mM KCl, 10 mM Tris-HCl pH 8.4. 1.5 mM MgCl_2_, 1 mg/mL gelatin), milli-Q water to a final volume of 15 μL and 20 ng of genomic DNA. Samples were subjected to 25 cycles of 94°C 1 min, 50°C 2 min, and 72°C 2 min and amplified products were visualized on agarose gels. PCR reactions were purified with the ExoSAP-IT^® ^(USB), following the manufacturer protocol. PCR products were cloned into pGEM^®^-T Easy vector system (Promega) and after colony screening, positive clones were sequenced for the ITS2 region. Sequencing reactions were performed using the DYEnamic™ ET Dye Terminator Cycle Sequencing Kit and a MegaBACE™ DNA analysis system (GE Healthcare Life Sciences).

Multiple sequences were obtained for each species (Table 1) depending on the PCR colony screening results and then further assembled. Sequences were analysed using PHRED/PHRAP/Consed [24] as well as by the Crossmatch software to trim vector sequences [25]. Sequence similarity searches were performed using the *blastx *program of the BLAST package against different databases [26]. Further comparisons have been performed between sequences described in the present work with those published elsewhere [23,27].

### Identification of *fbn9*

The identification of the *fbn*9 gene sequences of four neotropical anophelines (Table 1) was performed through PCR and sequencing analyses as follows. First, degenerate primers were designed based upon highly similar regions between two *fbn*9 homologs in *An. gambiae *(AGAP011197) and *Ae. aegypti *(AY432284.1) translated sequences available in public databases. PCR reactions were performed with 0.5 μM of each primer: 5fbn_deg4 (5'-AAYCARGCNCAYYTNGARAA-3') and 3fbn_deg4 (5'-CANCCICCICCRAAYTTNGTYTG-3') with the following parameters: 94°C 5 min, 15 cycles (94°C 30 sec, 50°C 30 sec, 72°C 1 min) where the annealing temperature was reduced by 1°C per cycle; followed by 20 cycles (94°C 30 sec, 50°C 30 sec 72°C 60 sec). Part of the amplified product was checked on agarose gel. In the absence of unspecific amplified bands, PCR products were re-amplified.

Fragments from the positive PCR reactions were purified using the QIAEX II^® ^Gel Extraction Kit (Qiagen) and cloned into pGEM^®^-T Easy vector system (Promega). Bacterial clones (*E. coli *- TOP10) were checked by PCR using the T7 (5'-TAATACGACTCACTATAGGG-3') and SP6 (5'-ATTTAGGTGACACTAG-3') primers (94°C 2 min, 35 cycles of 94°C 30 sec, 45°C 30 sec and 72°C 60 sec; followed by 5 min at 72°C). PCR products were sequenced as described above using T7 and SP6 primers. High quality consensus sequences were subjected to sequence similarity searches using the *blastx *program against an *An. gambiae *protein database built in the present work. The top BLAST hits to the *An. gambiae fbn9 *gene sequence (GenBank Gi: 167861677) were considered as potential orthologs for further analysis.

### Sequence analysis

All *fbn9 *gene sequences obtained from *An. aquasalis*, *An. darlingi*, *An. albitarsis*, and *An. nuneztovari *were aligned with ClustalX [28] and manually adjusted with BioEdit [29]. Analyses of the alignment coverage, the presence of conserved domains, the number of single-nucleotide polymorphisms (SNPs), the percentages of synonymous and non-synonymous substitutions, and the rates of transitions and transversions among the nucleotide sequences were performed through MEGA software [30].

For the studies on selection pressure, another multiple sequence alignment was built including other additional 60 *fbn9 *sequences from the *An. gambiae *complex deposited at the GenBank and described elsewhere [13]. Analysis of the alignments in terms of dN, dS, and dN/dS ratio were again performed with MEGA. To calculate the frequency of synonymous and non-synonymous substitutions, we applied the Nei-Gojobori method with the Jukes-Cantor correction [31,32].

### Phylogenetic analysis

For the phylogenetic analysis, a total of 21 *fbn*9 nucleotide sequences from 10 species have been selected that include: four partial sequences from *An. darlingi*, *An. aquasalis*, *An. nuneztovari*, and *An. albitarsis *(Amazon and Rondonia, Brazil) obtained in the present work together with other 16 sequences from six species belonging to the *An. gambiae *complex (Table 1). Multiple sequences from the same species, identified as A and B, refer to different alleles of the same specimen or different specimens collected.

The use of nucleotide sequences was chosen due to the high level of conservation observed in the amino acid sequences found in our species, which would result in less informative output. Both 5' and 3' ends from the *An. gambiae *sequences, which were not present in our sequences, were removed from the final alignment. The final alignment contained 365 sites, which corresponds to 42.9% of the *An. gambiae fbn9 *gene sequence.

Bayesian analysis with the Markov chain Monte Carlo (MCMC) sampling method as implemented in MrBayes (version 3.1) [33] has been performed. MCMC analyses were run as four chains (1 cold and 3 heated chains) for 1,500,000 generations, with sampling occurring every 1000 generations and with 25% of the initial samples discarded as "burn-in". The General Time Reversible (GTR) model assuming a gamma distribution with variation rate between the sites and an invariable proportion of the sites (GTR + inv + gamma) has been adopted. The sequence of *An. nuneztovari *was used as outgroup to construct the phylogenetic tree. Support values of the recovered trees were estimated as Bayesian posterior probabilities (pp). Well-supported clusters with the posterior probability of at least 0.80 were considered for data interpretation. The consensus tree was visualized and edited in FigTree, version 1.2 [34].

## Results

In the present study, partial nucleotide sequences of an important immune-related gene (*fbn9*) from four neotropical anopheline mosquito species have been obtained. Sequencing of the ITS2 region of the neotropical mosquito specimens allowed the identification of species with higher accuracy in addition to morphological identification [21]. Further comparisons of sequences from neotropical anophelines with publicly available sequences from the *An. gambiae *complex have been performed to apply selection pressure studies and phylogenetic analysis.

### Gene cloning and sequencing

**Figure 1:**
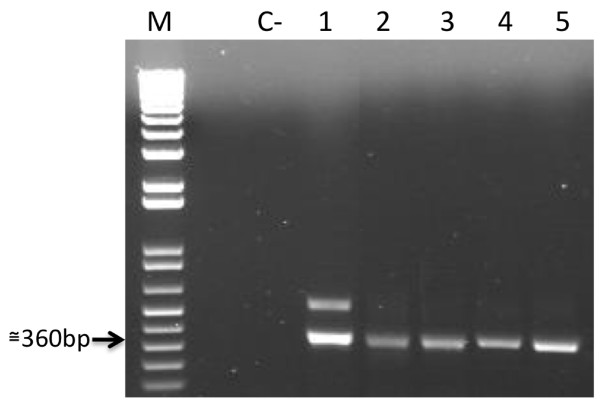
**Identification of *fbn*9 gene sequences**. Amplification of the *fbn*9 partial gene sequence from five neotropical anopheline mosquitoes: *An. aquasalis *(1), *An. marajoara *AM (see table 1) (2), *An. marajoara *RO (3), *An. darlingi *(4), and *An. nuneztovari *(5). Molecular weight (M, 1 kb plus ladder, Invitrogen) and negative control (C-) are indicated.

The *fbn*9 gene was identified through PCR in *An. aquasalis*, *An. darlingi*, *An. marajoara*, and *An. nuneztovari *mosquitoes (Figure 1). Results were confirmed through DNA sequencing followed by similarity searches using *blastx*. All sequences matching FBN9 protein sequences as the top hit with significant E-values and high percentage of identity (See additional file 1: Table S1). Sequences had around 360 nucleotides, corresponding to 42.5% of the gene, from positions 84 to 444 of the sequence (Gi: 167861677).

For *An. aquasalis*, a higher molecular weight band was detected (Figure 1) and sequenced, but no significant sequence similarity was detected through BLAST searches.

### Sequence analysis

**Figure 2:**
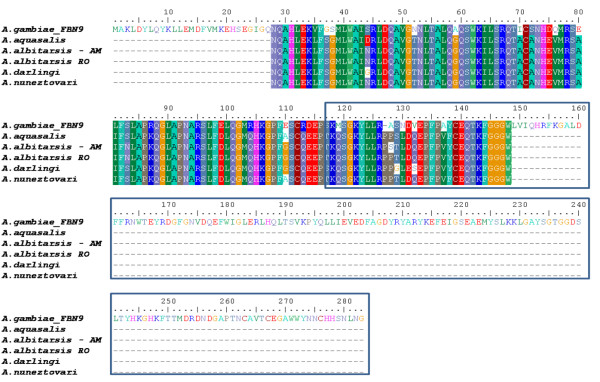
**FBN9 protein sequence analysis**. Alignment of predicted amino acid sequences of the FBN9 protein from different anopheline species using ClustalW. Sequence within box corresponds to the FREP domain conserved among FBN protein family members as described elsewhere.

The analysis of the sequences obtained for the neotropical mosquito species showed 79 SNPs in 360 nucleotides, where 11.33%correspond to the first, 7.59%to the second, and 81.01%to the third codon positions. Of the 72 polymorphic codons, 64 correspond to synonymous substitutions and eight are non-synonymous. In total, 61 transitions and 25 transversions were detected. When the *An. gambiae *complex sequences were included in the analysis, 128 nucleotide polymorphisms and 104 polymorphic codons were observed, resulting in 75 synonymous and 29 non-synonymous substitutions. A high degree of conservation among the amino acid sequences from anopheline mosquitoes has been found (Figure 2).

### Phylogenetic analysis

**Figure 3:**
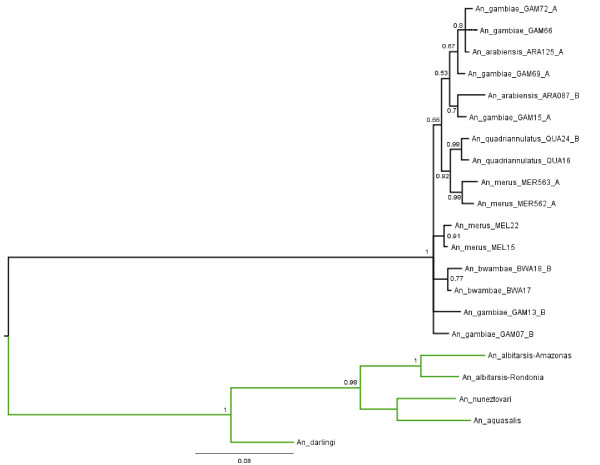
**Phylogenetic tree with 21 partial sequences of the *fbn9 *gene from 10 anopheline species (Table 1)**. Branches leading to the Brazilian (present study) and African anopheline species are indicated in green and black, respectively. Posterior probability values (pp) are indicated in this 50% majority-rule consensus (unrooted) tree. Letters A and B following the specimen abbreviation indicate two alleles of a single individual specimen as described elsewhere [13].

Phylogenetic relationships among 21 partial *fbn9 *gene sequences from 10 distinct anopheline species (Table 1) were reconstructed using a Bayesian approach as described above (Methods). The tree topology (Figure 3) suggests the presence of two well-supported sequence groups (pp = 1) corresponding to the African (black) and Neotropical (green) species analyzed in the present study. The former group includes different lineages of five species of the Gambiae complex with two lineages of *An. merus *(MER562_A and MER563_A) clustered together with other two of *An. quadriannulatus *(QUA16 and QUA24-B). Other relationships in the African group are also well-resolved considering the posterior probability cutoff (at least 0.80) adopted in our approach.

The phylogenetic relationships among the five neotropical species (green) are strongly supported by the analysis as showed in Figure 3. The two sequences of *An. albitarsis *(*An. marajoara*) originated from Amazon and Rondonia, Brazil, are closely related (pp = 1) and form a sister group with sequences of *An. aquasalis *and *An. nuneztovari *(pp = 0.98). The *An. darlingi *sequence seems to be more divergent across the Neotropical species. Further phylogenetic analysis supported the orthology prediction performed through sequence similarity-based searches aforementioned.

## Discussion

The unavailability of genomes from neotropical anopheline species hamper most of the studies related to function and evolutionary aspects of important genes in mosquitoes, which makes the use of alternative techniques the only available solution to overcome this problem. Recently, studies on differential gene expression of the coastal malaria vector *An. aquasalis*, infected with *Plasmodium vivax*, have been performed and three fibrinogen-related genes have been found, which differ from *fbn*9 [35].

In *An. gambiae*, FBN9 has been known to have an important function on the mosquito immune system, acting as a pattern recognition receptor. Its expression is up-regulated when mosquitoes are infected with bacteria and *Plasmodium *species [11]. The participation of this protein on the mosquito's interaction with parasites is very important because it determines the insect's vectorial competence [36].

This study showed the high conservation pattern present in this protein in both *An. gambiae *and in neotropical species belonging to the subgenus *Nyssorhynchus*, suggesting that this protein might have the same function in all these species. As recently stated, fibrinogen related proteins (FREPs), where FBN9 belongs, are part of the basal immune surveillance of mosquitoes by interacting with mosquito bacterial flora [11]. This may explain this conservation as all mosquito species ubiquitously harbour bacteria.

A comparison of synonymous and nonsynonymous substitution rates in protein coding genes provides an important means for understanding molecular evolution [37]. When comparing the sequences in different Anopheline species, a higher frequency of polymorphic sites on the third base of the codon reflects the higher presence of synonymous substitutions. The results presented here are in concordance with previous work analyzing the patterns of molecular evolution in the *fbn9 *gene using 60 sequences of six species from the Gambiae complex [13]. Here, synonymous substitutions are in higher frequency than non-synonymous substitutions resulting in a *dN*/*dS *< 1 ratio, which corresponds to a negative or purifying natural selective pressure, perhaps limiting alterations at protein level. This observation suggests that this region may be important to maintaining the function and/or the structure of this protein. Recently, Lehmann and colleagues studied four immunity-related genes (SP14D1, GNBP, defensin, and gambicin) in *An. gambiae *and found no evidence to prove that selection was mediated by pathogens that are transmitted to humans [38].

The indication of conserved function of the *fbn9 *gene between the Gambiae complex and the Brazilian anophelines is interesting if one considers that the separation of sub-genus *Cellia *and *Nyssorhynchus *occurred around 94 my ago, based on mtDNA analysis [39]. Further studies on the expression of *fbn9 *in Neotropical mosquito species and its role during infection with *Plasmodium *species will provide further information on this important immune-related gene.

## Conclusions

In the present work, the *fbn*9 gene sequences of four neotropical anopheline species have been compared with their homologs in the *An. gambiae *complex to gain insights into insect immunity. Sequence analysis shows a high degree of conservation of the *fbn*9 gene in all species compared and suggests that these sequences are under negative selection pressure. Bayesian phylogenetic analysis supports the hypothesis of neotropical anophelines (subgenus *Nyssorhynchus*) and African mosquitoes (subgenus *Cellia*) forming two well-supported clades. The present work suggests a possible conserved role for *fbn*9 in the immune response of a broader range of anopheline species. Identification of the genes involved in the mosquito immune response to parasite infections shall allow the design of novel pathogen control strategies.

## Abbreviations

MCMC: Markov chain Monte Carlo; NCBI: National Center for Biotechnology Information; PCR: Polymerase Chain Reaction. PCR primer abbreviation: 5fbn_deg4: Forward fbn degenerated primer 4; 3fbn_deg4: Reverse fbn degenerated primer 4; CP16: Forward rDNA ITS2 region primer; CP17: Reverse rDNA ITS2 region primer; T7: T7 universal primer; SP6: SP6 universal primer.

## Competing interests

The authors declare that they have no competing interests.

## Authors' contributions

SBO participated in the study design and carried out the experiments. ICI helped on the cloning and sequencing. WPT collected and identified mosquito specimens. JCR helped on sequences analysis. LAN designed and coordinated the phylogenetic analysis and co-wrote this manuscript. CFAB helped in the study design and in all experimental phases. LAM conceived and coordinated the study and drafted the manuscript. All authors read and approved the final manuscript.

## Supplementary Material

Additional file 1 **Blast analysis of the FBN9 translated sequences**. *Blastx *analysis of the FBN9 translated sequences against the top hit from *A. gambiae *protein database (FBN9 - Gi: 167861677) Click here for file
