# Supplementary material for: Identification of a fibrinogen-related protein (FBN9) gene in neotropical anopheline mosquitoes
Source: Malar J. 2011 Feb 2;10:21. doi: 10.1186/1475-2875-10-21 (PMC3055219; doi:10.1186/1475-2875-10-21)
Supplement: Additional file 1 — Blast analysis of the FBN9 translated sequences. Blastx analysis of the FBN9 translated sequences against the top hit from A. gambiae protein database (FBN9 - Gi: 167861677) [file 1475-2875-10-21-S1.DOC]

**Additional table S1.** *Blastx* analysis of the FBN9 translated sequences against the top hit from *A. gambiae* protein database (FBN9 – Gi: 167861677)

| **Species** | **Identity** (**%)** | **Coverage** | ***e-value***^a^ | ***Score*** |
| --- | --- | --- | --- | --- |
| *An. aquasalis* | **78.51** | **120** | **1.00E-53** | 200 |
|  | 45.79 | 107 | 3.00E-18 | 87 |
|  | 43.52 | 108 | 1.00E-14 | 75.5 |
| *An. darlingi* | **78** | **121** | **7.00E-53** | 202 |
|  | 46 | 107 | 3.00E-18 | 87.0 |
|  | 44 | 108 | 8.00E-15 | 75.9 |
| *An. marajoara*_AM | **78** | **121** | **2.00E-53** | 203 |
|  | 46 | 107 | 9.00E-19 | 89.0 |
|  | 44 | 108 | 4.00E-15 | 77.0 |
| *An. marajoara*_RO | **77** | **121** | **9.00E-53** | 201 |
|  | 45 | 107 | 3.00E-18 | 87.0 |
|  | 44 | 108 | 4.00E-15 | 77.0 |
| *An. nuneztovari* | **77** | **121** | **9.00E-53** | 201 |
|  | 44 | 107 | 6.00E-18 | 86.3 |
|  | 43 | 108 | 6.00E-15 | 76.3 |

^a^ Three results from *blastx* with highest score and lowest E-value
